# Tumor-to-tumor spread: a case report and literature review of renal cell carcinoma metastasis into thyroid cancer

**DOI:** 10.1186/s12957-023-03220-5

**Published:** 2023-11-22

**Authors:** Cassidy Gawlik, Jason Lane, Mark Horattas

**Affiliations:** 1 Department of General Surgery, Cleveland Clinic Akron General, Akron, OH USA; 2 Department of Pathology, Cleveland Clinic Akron General, Akron, OH USA

**Keywords:** Tumor-to-tumor metastasis, Thyroid carcinoma, Renal cell carcinoma

## Abstract

Tumor-to-tumor metastasis is a rare, yet important entity. Patients with a history of renal cell carcinoma (RCC) may have tumor deposits to the thyroid gland preceding or following their initial cancer diagnosis for many years. The diagnosis can be challenging, and clinicians must remain suspicious of a newly found thyroid nodule in a patient with a history of RCC. In this review, we report a case of a patient with RCC who was incidentally found to have a thyroid nodule on surveillance imaging found to be consistent with tumor-to-tumor metastasis from RCC into papillary thyroid carcinoma. It is imperative to consider this diagnosis as the thyroid is the most common site of spread, and treatment with partial or total thyroidectomy has led to improved survival.

## Background

The thyroid gland is highly vascular, and one might presume it to be a common site for metastatic disease [1]. Paradoxically, metastatic spread to the thyroid is rare, and only 2% of all thyroid malignancies are metastases [1]. Breast cancer was historically thought to be the most common source, but now renal cell carcinoma (RCC) encompasses 48% of all metastatic disease to the thyroid [2]. An even rarer, yet important phenomenon is tumor-to-tumor metastasis. An increasing number of cases are being reported in the literature, and RCC is also the most common donor cancer to deposit in thyroid cancer. This concept should not be overlooked in patients presenting with a new thyroid nodule and a history of RCC because improved survival has been shown with resection of metastatic disease [1]. Here, we describe a case in which a patient underwent a nephrectomy for RCC and was subsequently found to have a thyroid nodule on surveillance imaging 1 year later consistent with metastatic RCC into follicular variant of papillary thyroid carcinoma (FVPTC).

## Case presentation

**Fig. 1:**
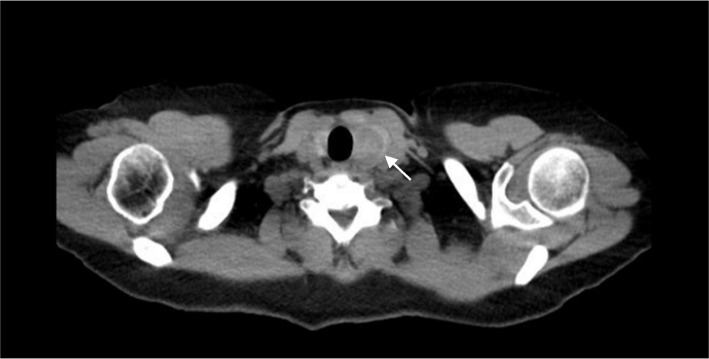
CT chest axial view. Arrow denotes 2.6 cm  left thyroid nodule

**Fig. 2:**
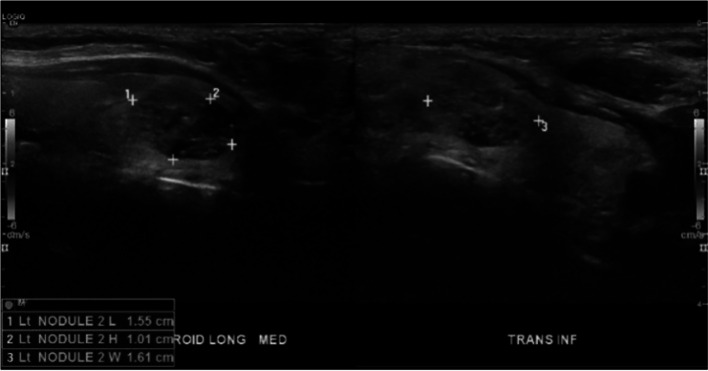
Thyroid US revealing 1.55 × 1.01 × 1.61 cm nodule with corresponding cytology diagnosis of atypia of undetermined significance

**Fig. 3:**
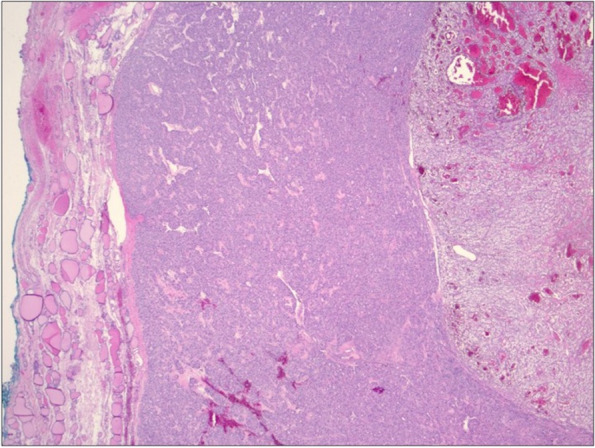
H&E stain showing a rim of non-neoplastic thyroid (left), FVPTC (center), and metastatic clear cell RCC (right) (20 × total magnification)

**Fig. 4:**
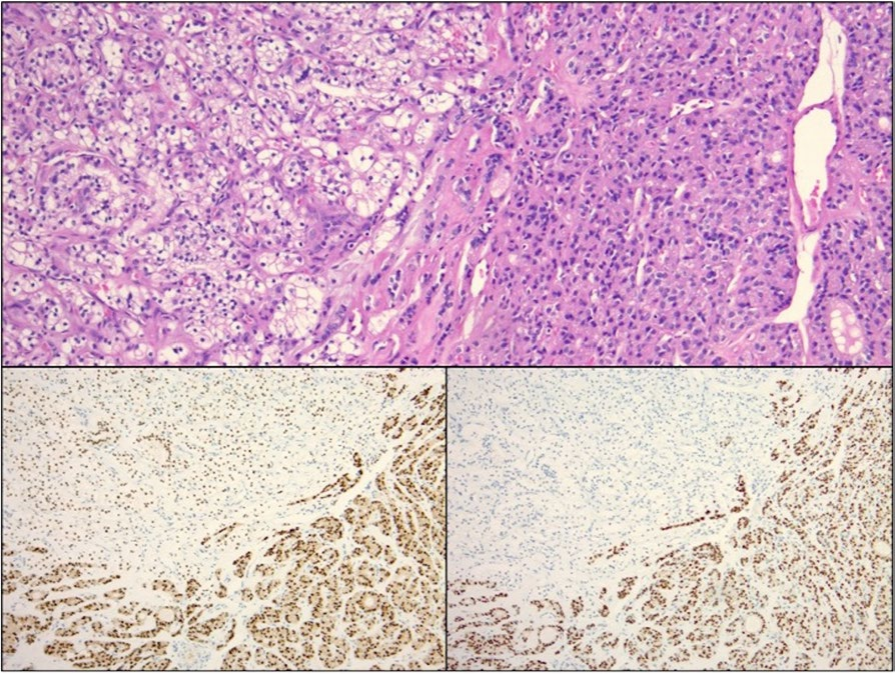
H&E stain section (upper panel of image, 100 × total magnification) showing juxtaposition of clear cell RCC (left) and FVPTC (right). Immunohistochemical stain for PAX8 (lower left panel) shows expected positivity in both the RCC and the papillary thyroid carcinoma, while immunostain for TTF-1 (lower right panel) shows differential expression with positivity in the papillary thyroid carcinoma and negativity in the RCC

Our patient is a 63-year-old female who initially presented to her primary care physician in 2021 with epigastric abdominal pain. A computed tomography (CT) abdomen and pelvis was ordered which incidentally found a 6.5 × 3.0 x 5.9 cm exophytic renal mass concerning for malignancy. Her past medical history was significant for anxiety, asthma, migraines and restless leg syndrome. Surgical history included a cesarean section, tubal ligation, and right thyroid lobectomy in 1995, which the pathology resulted as benign. Her family history was significant for a father with unspecified kidney disease but negative for malignancy. She denied alcohol or tobacco use. She was then referred to urology in 2021, who performed a CT urogram which revealed the same 6-cm mass most consistent with renal cell carcinoma along with an indeterminate hepatic hypodensity. She did not note any hematuria or flank pain. Her creatine was 0.65 mg/dL (0.58–0.96 mg/dL), BUN 14 mg/dL (7–12 mg/dL), and eGFR > 60 mL/min (> 60 mL/min), and her liver function tests were within normal limits. One month later, she underwent a right partial nephrectomy along with liver biopsy and ablation. Pathology reports revealed a 6-cm grade 3 clear cell renal cell carcinoma with negative surgical margins and no lymphatic involvement consistent with stage pT1b RCC as well as benign hepatic parenchyma. As part of surveillance imaging, she had a CT chest 1 year later which was negative for suspicious pulmonary nodules but included findings of a 2.6-cm hypointense left thyroid nodule (Fig. 1) with coarse calcifications. This was unchanged from prior CT imaging in 2019. However, an ultrasound (US) of the thyroid gland was performed for further evaluation, and this revealed a stable 2.6 × 2.6 × 2.0 cm left middle thyroid lobe nodule graded TI-RADS4, which is a radiologic grading system used to predict the risk of thyroid cancer based on ultrasound features. Two additional mixed cystic and solid nodules, taller than wide with smooth margins and punctate echogenic foci graded TI-RADS4, were also noted to measure 1.55 × 1.01 × 1.61 cm (Fig. 2) and 1.5 × 1.03 × 1.39 cm, respectively. Ultrasound-guided fine needle aspiration (FNA) of the latter two nodules was performed, and the cytology report of the former nodule was consistent with atypical cells of undetermined significance with architectural atypia, and the latter nodule was benign. During this procedure, the isthmus was also found to have a new 1.1-cm complex nodule and microcalcification for which short follow-up was recommended. She was then referred to general surgery. The patient denied any symptoms relating to her thyroid, and on exam, she had a palpable fullness of her left thyroid gland. Thyroid function tests were not performed. Given the indeterminate FNA results, Afirma™ gene expression classifier testing was pursued which resulted back as “suspicious” with a 50% risk of malignancy. Genetic testing for RET/PTC and BRAFV600E mutations were  negative. The patient then underwent a left thyroid lobectomy and isthmectomy. Pathology report revealed a 1.5-cm adenomatoid nodule and 2.5-cm encapsulated follicular variant of papillary thyroid cancer with multiple foci of metastasis of clear cell RCC, specifically to the thyroid cancer (Fig. 3). Immunohistochemical (IHC) staining revealed PAX-8 positivity for both the RCC and FVPTC component, and thyroid transcription factor-1 (TTF-1) and thyroglobulin (TG) both stained positive in the papillary thyroid cancer tissue (Fig. 4). Lymph nodes were negative for disease, and pathologic staging for the thyroid carcinoma was pT2 N0. Given this unexpected finding of tumor-to-tumor disease, the patient was referred to oncology. Currently, there are no plans for adjuvant therapy, and she is undergoing yearly CT imaging of the chest, abdomen, and pelvis and MRI of the brain. At this point in time, she does not have any evidence of further disease.

## Discussion

Renal cell carcinoma represents 90% of all renal tumors and 3% of adult malignancies [3]. The classic findings of flank pain, hematuria, and a palpable mass are only present in 10% of patients, and most are diagnosed after incidental imaging findings [4]. Survival outcomes are dependent on the stage at diagnosis, and treatment typically involves nephrectomy for curative intent. It is estimated the 30% of patients have metastatic disease at the time of diagnosis, and that 20–50% of patients develop metastatic disease following nephrectomy with curative intent [4]. Metastatic RCC portends a poor prognosis with a median overall survival of 2 years and a 5-year survival rate of 12% [1]. However, outcomes are greatly improved when solitary metastatic lesions can be resected, but this is only present in 3% of those with metastatic disease [1]. With isolated metastasis to the thyroid gland, 5-year survival increases to 50% [1]. Other favorable sites of RCC spread include the lungs, regional lymph nodes, bone, head, and neck region. RCC is the most common malignancy that metastasizes to the thyroid, and it is responsible for over 50% of all clinically recognizable metastasis to the thyroid [1].

Thyroid metastases can technically occur from any organ in the body. Common sources include tumors from the colon, rectum, lung, and sarcoma. Yet, RCC is the most prevalent primary cancer comprising 48% of all metastatic disease [2]. One unique concept that deserves special attention is tumor-to-tumor metastasis. Albeit rare, RCC is also the most common donor of tumor-to-tumor metastasis within the thyroid [5]. This concept of tumor-to-tumor metastasis was first reported in 1902 by Berent et al., cited by Gowda et al., in which a 58-year-old white male with squamous cell carcinoma of the lower jaw had tumor metastasis into a RCC [6]. This theory abides by strict criteria established by Dobbing and by Campbell et al. in 1968 that include the following: two or more distinct tumors, with the recipient being a true neoplasm, and a true metastatic deposit within the neoplastic tissue of the recipient tumor. Furthermore, this definition excludes collision tumors, lymphovascular spread without actual invasion, and metastasis into hematopoietic malignancies [3, 7]. Interestingly, RCC can act as both a donor and recipient. Other frequent donor malignancies are from the breast, lung, and prostate, while the most common receptor tumors are papillary thyroid cancer and meningiomas [8]. Of the 150 cases of tumor-to-tumor metastasis reported in the English language literature, 29 involve a thyroid tumor as a recipient neoplasm. To the best of our knowledge, 14 cases were found to involve RCC metastasizing to thyroid cancer and are summarized in Table 1 with our patient being the 15th case [6, 9]. Of all the thyroid pathologies, RCC favors spread mainly to follicular variant of papillary thyroid cancer, in addition to follicular adenoma and Hürthle cell adenoma [6].

**Table 1:** Cases of tumor-to-tumor metastasis from RCC to thyroid carcinoma

Author	Age	Gender	Recipient thyroid neoplasm	Size of thyroid neoplasm	Time interval to diagnosis of metastatic disease	IHCresults of metastatic RCC	Treatment
Badawi et al. [3]	63	F	FVPTC	Multinodular, largest 1.9 cm	14 years	CD10, PAX-8, CAIX, galectin-3, and vimentin ( +)	Total thyroidectomy
						TG, TTF-1, CK7, CK19 ( −)	
Kefeli et al. [5]	80	F	FVPTC	4 cm	18 years	PAX-8, CD10, vimentin ( +)	Left thyroidectomy
						TG, TTF-1, GATA-3, PTH hormone, chromogranin-A, CEA, calcitonin ( −)	
Manini et al. [8]	42	M	FVPTC	5 cm	Synchronous	CD10, PAX-8, and CAIX ( +)	Total thyroidectomy
Medas et al. [9]	62	F	FA	Multinodular, largest 5.3 cm	6 years	CD10 ( +)	Total thyroidectomy
						TG, TTF-1, galectin-3 ( −)	
Qian et al. [10]	53	F	Hürthle cell adenoma	2 cm	Synchronous	Vimentin, CD10 ( +)	Total thyroidectomy
						TG, TTF ( −)	
Baloch et al. [11]	78	M	FVPTC	Multiple nodules, size not reported	2 years	NA	NA
Bohnv et al. [12]	68	M	Papillary thyroid carcinoma	2.5 cm	2 years	RCC ( +)	Total thyroidectomy
						TG ( −)	
Koo et al., cited by Bohn [12]	48	F	FA	3 cm	5 years	Keratin (not further specified), CD10, galacetin-3 (+)	NA
						TG, TTF-1, calcitonin, inhibin ( −)	
Rosai et al., cited by Bohn [12]	NA	NA	FA	NA	NA	TG ( −)	NA
Ryska et al. [13]	52	M	Oncocytic Hürthle cell carcinoma	Multinodular, largest 2.5 cm	13 months	Keratin AE1/AE3, vimentin, EMA ( +)	Total thyroidectomy
						TG, CEA, calcitonin, cytokeratin 19 ( −)	
Wolf et al. [14]	60	F	FA	3 cm	2 years	NA	Left lobectomy
Yu et al. [15]	61	M	FVPTC	Multinodular largest 6 cm	3 years	Keratin CAM 5.2, CD10, vimentin( +)	Total thyroidectomy
						TG, TTF-1, RCC, p63 ( −)	
Ghossein et al. [16]	65	F	FVPTC	1.1 cm	− 2 months^a^	NA	Thyroid lobectomy
Chacho et al. [17]	72	F	FA	2 cm	Synchronous	NA	NA
Gawlik et al.	63	F	FVPTC	2.5 cm	13 months	PAX-8 ( +)	Left lobectomy
						TG, TTF-1, chromogranin, synaptophysin ( −)	

*IHC* immunohistochemistry, *FVPTC* follicular variant papillary thyroid cancer, *FA* follicular adenoma, *TG* thyroglobulin, *TTF-1* thyroid transcription factor-1, *CAIX* carbonic anhydrase IX, *EMA* epithelial membrane antigen, *NA* not available

^a^Time interval of negative indicates metastasis to the thyroid was diagnosed before the primary RCC

The thyroid gland and kidney have a well-known interrelationship. The thyroid gland is necessary for renal cell growth and maintenance of hydro-electrolyte homeostasis [18]. On the other hand, the kidney is responsible for elimination of thyroid hormones and regulation of their serum levels. Thus, dysfunction in one system can affect the metabolism of the other organ. Many factors connecting the thyroid gland and kidney have been studied to hypothesize why tumors spread preferentially between the two organ systems. Specific risk factors include obesity, family history, male sex, and increasing age, and these are shown to increase the risk of both cancers [18]. Furthermore, an individual’s genetic makeup may increase susceptibility to both cancers. An example of this can be seen in those with the PTEN germline mutation. This can be present in patients with or without a genetic disease known as Cowden syndrome, which leads to a variety of benign and malignant tumors in the breast, thyroid, colon, and renal system. One study demonstrated that thyroid cancer of follicular origin and renal cancer have been found in great frequency in those with the PTEN germline mutation [18]. Even people without Cowden syndrome who express the PTEN mutation are still at higher risk for thyroid carcinoma and RCC [18]. Clinicians should be aware of this relationship and keep in mind the risk of developing a secondary malignancy in those with thyroid or renal cancers.

It is thought that the thyroid is an uncommon site for metastasis due to fast arterial flow and high oxygen saturation and iodine concentration that inhibits the growth of malignant cells [18]. However, when disrupted, some believe the abnormal iodine and oxygen concentration becomes a nidus for metastases [19]. Although the pathophysiologic mechanism of spread is up for debate, most authors accept one of two proposed theories. In the mechanical theory, the development of metastasis depends on the number of viable tumor cells reaching recipient neoplasm via the rich vascular supply of the tumor tissue [6]. The rich lymphovascular network seen in RCC allows deposition of metastatic tumor cells within the thyroid gland [9]. The tumor cells are thought to travel along the venous plexus of Batson, allowing bypass of the lungs [10]. On the other hand, the seed and soil theory supports that metastatic development occurs when a compatible environment, the soil, is provided to viable tumor cells, the seed [6]. The biochemical contents of high lipid and glycogen found in RCC provide these cells fertility to then flourish in the rich blood supply of the thyroid leading to growth of metastatic deposits [6, 11].

Diagnosing RCC metastasis into the thyroid can be a challenge. There have been no documented cases in which a preoperative diagnosis of tumor-to-tumor metastasis has been made by FNA. Thus, clinical suspicion must remain high. First, the clinical incidence is rare, estimated to be 0.36%, and is more commonly seen in autopsy specimens, with reported incidence ranging from 1.9 to 24% [2]. Even when a patient presents with a new thyroid nodule and a clinical history of malignancy, the diagnosis is most likely a benign lesion [20]. Moreover, symptoms present on a spectrum and can range from asymptomatic to rare reports of dysphonia from mass effect on the recurrent laryngeal nerve or even airway compromise from compression of the trachea [2]. Several studies have found that patients are more likely to present with symptoms when lesions are larger than 4 cm [21]. Thyroid function tests are usually within normal limits, and imaging findings can share features of both benign and malignant disease [2, 21]. Regarding ultrasound imaging features, one study suggested hypoechoic solid nodules, and increased vascularity should clue one toward a diagnosis of metastasis [22]. Additionally, metastasis of the thyroid gland can be synchronous or metachronous and either precede or follow the diagnosis of RCC by many years [23]. The average latency period is between 6 and 12.5 years after resection of a primary RCC but has been reported to be as long as 31 years [21, 22]. The diagnosis of a metastatic deposits may not be possible with clinical findings, and thus, pathologists become key players.

Despite the unique histopathologic features seen under the microscope, metastatic RCC can morphologically simulate primary thyroid neoplasms [10]. This is seen regarding the clear cell appearance seen in both well-differentiated thyroid carcinoma and RCC [24]. This poses a challenge to the pathologist because one must be able to exclude other primary thyroid lesions that exhibit clear cell change, such as follicular neoplasms (Hürthle cell type), papillary carcinomas, medullary carcinomas, and paragangliomas [20]. It then becomes the pathologist’s responsibility to identify other histopathological patterns suggesting tumor-to-tumor metastasis, even in the absence of comprehensive patient information [5]. One of these features that should heighten suspicion that tumor-to-tumor metastasis may be present is the identification of a distinct, dimorphic histological pattern with an abrupt transition between the two [12, 20]. When the two cell populations differ greatly, as in the example of a gastric adenocarcinoma metastasizing into a lipoma, this pattern is obvious. On the contrary, when there is a small tissue sample size involving a large benign tumor with a small donor seed, it may not be easy to differentiate two cell populations [8]. Further complicating the diagnosis is the situation of two carcinomas having similar cytoarchitectures. When one is viewed inside the other, as seen in RCC metastasizing to the thyroid, pathologists are faced with a quandary [8]. Another feature that can be relied upon for proper diagnosis is a multifocal growth pattern and sinusoidal patterns of vascularity [3, 24]. Close attention should be paid to intraluminal erythrocytes or the so-called “bloody follicles” which are suggestive of metastatic RCC [5]. Moreover, most metastatic neoplasms lack or display a minimal amount of reaction (desmoplastic, inflammatory, or myxoid) of the recipient tumor to metastatic deposits [6]. Along with these features, it must not be forgotten how valuable it may be to compare current slides with the patient’s prior nephrectomy material [24]. Unfortunately, this type of comparison is not always readily available, and this is when further testing with immunohistochemical stains to clarify the diagnosis may be necessary [24]. Nonetheless, pathologists should have a high suspicion of tumor-to-tumor metastasis if any of these above characteristics are identified.

The addition of immunohistochemical staining usually gives way to the identity of the origin of cell populations [5]. Primary thyroid tumors of follicular origin stain positive for thyroglobulin (TG) and thyroid transcription factor-1 (TTF-1). On the contrary, clear cell RCC stains positive for a variety of markers such as vimentin, galectin-3, CD10, CAIX, renal cell carcinoma (RCC), PAX-8, and epithelial membrane antigen (EMA) [3, 24]. However, caution should be taken with the use of PAX-8 because it can be expressed in both thyroid and renal neoplasms as well as other tumors gynecologic origin. Thus, a panel of stains must be used to guide the diagnosis. It has been suggested that positivity for CAIX and PAX-8 and negativity for TTF-1, TG, and keratin 7 are supportive of metastatic RCC when found in the thyroid gland [13]. Our patient’s staining pattern was PAX-8( +)/TG( −)/TTF-1( −)-supported RCC as the origin of the tumor. In a series that included 12 metastatic clear cell RCC to the thyroid, Cimino-Mathews et al. found that TTF-1( −)/TG( −)/CAIX( +) was 100% sensitive and 100% specific for metastatic clear cell RCC [13, 25]. With the use of a combination of stains from both thyroid and renal origin, the pathology can be confidently concluded.

In general, metastatic RCC carries a poor prognosis, with a median overall survival of 2 years and a 5-year survival rate of 12% [4]. In those with disseminated disease, overall survival is noted to be better with spread to the thyroid and other glandular organs such as adrenals, pancreas, and salivary glands, when compared with other solid organs [1]. One case series has shown that the median survival time after diagnosing thyroid metastasis from RCC is 54 months [2]. One study by Alt et al. reported that complete resection of all metastatic disease, regardless of organ sites, improved cancer-specific survival when compared to those without complete surgical resection [1, 26]. Literature has shown a 5-year survival rate of 40% regardless of the type of thyroid surgery [1]. No official treatment guidelines exist for RCC metastasis into the thyroid gland. A multidisciplinary approach must be taken, and surgical resection of the thyroid is currently accepted as the best treatment for RCC metastasizing to the thyroid [27]. Whether to perform a total versus partial thyroidectomy is still up for debate, and research has failed to prove a difference in outcomes between the two operations [21]. However, the goal of surgical resection should be to remove the site of metastasis to prevent aggressive neck disease [1]. Fortunately, our patient was able to have all metastatic disease resected, and surveillance imaging has remained negative for recurrence. Although surgery can improve outcomes, the main cause of death in patients with metastatic RCC is due to disseminated disease. The advent of immunotherapy has advanced the treatment of this disease and has been shown to prolong overall survival and progression-free survival [21]. This promising therapy may provide further treatment options for those with metastatic disease and can provide hope to many in the future.

## Conclusion

Tumor-to-tumor metastasis is a rare yet interesting phenomenon, and when the thyroid is involved, RCC is the most common cancer found. In this article, we describe a case report of a patient who had an incidental finding of a thyroid lesion discovered on surveillance imaging for RCC that resulted as metastatic RCC. These deposits can be discovered at the initial cancer diagnosis or can take decades to present. The thyroid and kidney have a special relationship, and disruption in one organ system can alter the other. Thus, newly found thyroid nodules in a patient with a history of RCC should not be overlooked, and appropriate workup should be pursued with a tissue diagnosis. Immunohistochemical staining is vital to reaching the diagnosis. Fortunately, the thyroid is a favorable site of metastasis, and overall survival is improved with resection of metastatic disease. No official guidelines exist for the management of this disease, but we support total or subtotal thyroidectomy with the goal of removing all metastatic deposits and the aim of prolonging the patient’s life.

## Data Availability

Data sharing is not applicable to this article as no datasets were generated or analyzed during the current study.

## Abbreviations

RCC: Renal cell carcinoma
FVPTC: Follicular variant of papillary thyroid carcinoma
CT: Computed tomography
US: Ultrasound
FNA: Fine needle aspiration
TTF-1: Thyroid transcription factor-1
TG: Thyroglobulin
IHC: Immunohistochemistry
FA: Follicular adenoma
CAIX: Carbonic anhydrase IX
